# Epidemiology of Vascular Access in Patients Undergoing Chronic Hemodialysis Treatment in Greece

**DOI:** 10.3390/jcm14134571

**Published:** 2025-06-27

**Authors:** Athanasios Nousis, Maria Tziastoudi, Niki Oustampasidou, Maria Efthymiadi, Maria Divani, Theodoros Eleftheriadis, Ioannis Stefanidis

**Affiliations:** Department of Nephrology, Faculty of Medicine, School of Health Sciences, University of Thessaly, 41500 Larissa, Greece; thanosnousis@gmail.com (A.N.); nzorz@uth.gr (N.O.); mariaeuthimiadi@hotmail.gr (M.E.); mariadivani@yahoo.com (M.D.); teleftheriadis@yahoo.com (T.E.); stefanid@med.uth.gr (I.S.)

**Keywords:** vascular access, hemodialysis, arteriovenous fistula, chronic kidney disease, survival analysis, quality of life

## Abstract

**Background:** Vascular access (VA) is one of the most critical procedures during dialysis for patients with end-stage renal disease (ESRD), as it influences morbidity, mortality, and quality of life. **Methods:** This cross-sectional study analyzed the vascular access epidemiology of patients undergoing chronic HD in 15 nephrology centers across Greece from 2013 to 2019. Data on VA type, demographic characteristics, fatigue severity, and quality of life were gathered from a sample of 373 patients. **Results:** The prevailing result of this study is that arteriovenous fistula (AVF) was the commonly practiced VA, and its associated survival outcomes were better when compared to arteriovenous grafts (AVGs) and central venous catheters (CVCs). Patients with AVFs had significantly longer survival times (median 165 months) compared to non-fistula access. Furthermore, the degree of fatigue and quality of life were also dependent on the type of VA used, with patients on AVF having lower fatigue levels and better quality of life. Age, gender, and an early nephrologist referral were noted to affect the selection and the rate of maturation of VA. Despite AVF being the preferred VA, late referrals and high initial reliance on CVCs remain challenges. **Conclusions:** This study underscores the need for early nephrological intervention, surveillance programs, and patient education to optimize vascular access outcomes. Future research should focus on national strategies to reduce CVC-related complications and improve long-term HD care in Greece.

## 1. Introduction

Chronic kidney disease (CKD) impacts 14.9% of the U.S. population, with the highest prevalence observed among females (16%), adults over 65 years old (38.6%), Black individuals (17%), and Hispanics (15.3%) [1,2]. Hemodialysis (HD) is the most common form of renal replacement therapy, implemented in 70% to 90% of patients, all of whom require vascular access for the procedure [3]. However, selecting the most appropriate type of vascular access (VA) remains a challenge to date [4].

**Arteriovenous fistulas (AVFs)** [5], **arteriovenous grafts (AVGs)** [6], and **central venous catheters (CVCs)** are the main types of vascular access [7]. Among these, clinical guidelines suggest AVFs because of their lower rates of complications and better survival outcomes, as catheters are linked to increased risks of mortality, infection, and cardiovascular events [8]. Patients with a functioning AVF experience the lowest overall risk, although recent studies indicate that variations in comorbidities associated with different catheter types may partially explain these outcomes [9,10]. CVCs are typically used in urgent situations or when permanent access is not yet established, but they carry a higher likelihood of infection and thrombosis [11].

Despite ongoing efforts to increase the use of permanent vascular access and minimize the risks linked to tunneled dialysis catheters (TDCs), approximately 80% of patients in the United States start hemodialysis (HD) with a TDC [1]. Data from the United States Renal Data System (USRDS) show that in 2016, 80% of patients began HD with a catheter, and 69% were still using a catheter 90 days after initiation. Over the period from 2005 to 2016, AV fistula use at HD initiation increased from 12% to 17%, while the percentage of patients either using or maturing an AV fistula at initiation rose from 28.9% to 33%. At the start of dialysis, 17% of patients exclusively used an AV fistula, which increased to 64% after one year and 71% after two years. It is noteworthy to mention that the proportion of prevalent HD patients using an AV fistula rose from 32% in 2003 to 63% by 2014 [12]. The use of AV grafts was relatively low, with 3% of patients using one at HD initiation, increasing to 15% at one year and 17% at two years. By one year after HD initiation, 79% of patients were using either an AV fistula or AV graft without a catheter, rising to 88% by the two-year mark. As of May 2017, 62.8% of prevalent dialysis patients were using an AV fistula. However, of AV fistulas placed between June 2014 and May 2016, 39% failed to mature adequately for dialysis use, and among those that did, the median time to first use was 108 days.

Globally, the initiation of dialysis often involves the use of temporary and tunneled catheters, particularly in regions where there is a limited availability of vascular access specialists. More specifically, a study leveraged data from 167 countries and found that in 31 countries, more than 75% of patients began hemodialysis (HD) using a temporary catheter. Two of these countries were in Eastern and Central Europe, and four were in Western Europe. In contrast, seven countries reported that over 75% of patients started HD with arteriovenous fistulas or grafts. Another seven countries had more than 75% of patients initiating HD with tunneled dialysis catheters. Tunneled dialysis catheter use for HD initiation was higher than the global median in Africa (10%), North America and the Caribbean (22%), and Oceania and Southeast Asia (OSEA) (7%). Among low-income countries (LICs), 57% had over 75% of patients starting HD with a temporary catheter, compared to only 5% of high-income countries (HICs) [13].

Another study found that patient demographics played a role in AV fistula success, with younger patients experiencing higher maturation rates and shorter time to use. Males had higher maturation rates and shorter time to first use compared to females, while Black patients had the highest AV fistula maturation failure rates among racial groups [14]. It is noteworthy to mention that several comorbidities affect vascular access outcomes. For instance, cardiovascular comorbidities and diabetes mellitus, as well as older age, make the creation and maintenance of a well-functioning vascular access more difficult [15].

The latest Kidney Dialysis Outcomes Quality Initiative (KDOQI) guidelines emphasize patient-centered care through the ESKD Life-Plan, which guides kidney replacement therapy decisions [16]. Developed collaboratively with a multidisciplinary team, the Life-Plan considers treatment modality (HD, PD, or transplantation), setting (home or center), vascular access type, patient health, preferences, anatomy, and disease stage [17].

Recognizing the importance of creating and maintaining functional vascular access, which significantly affects the effectiveness of treatment, and given the fact that there are no data for the Greek population on vascular access types in hemodialysis, this study initially examined the frequency and types of vascular access in Greece and its association with early nephrological referral. In addition, another aim was to investigate the influence of vascular access on the quality of life, morbidity, and mortality of hemodialysis patients. Finally, a survival analysis by type of vascular access was also conducted.

## 2. Materials and Methods

We conducted a cross-sectional study on patients with end-stage renal disease (ESRD) undergoing hemodialysis in 15 nephrology centers across Attica. The participants were consecutively selected from Extracorporeal Dialysis Units (EDUs) of public hospitals, EDUs in the private sector, and Chronic Hemodialysis Units (CHUs) between January 2013 and December 2019. The exclusion criteria included limited communication ability and hospitalized patients.

This study obtained approvals from the Ethics Research Board of the University of Thessaly and permissions from the scientific councils of the hospitals where data were collected, and questionnaires were distributed. The participants were also informed about their voluntary participation, data confidentiality, and the secure handling of collected information. The participants were given an informed consent form. Τhe survival data were collected prospectively. Regarding censoring, the primary endpoint was all-cause mortality.

Several variables were recorded per patient, such as demographic characteristics, primary kidney disease, comorbidities, type of vascular access, assessment by nephrologist, survival data, and questionnaires about quality of life (QoL), fatigue, and restless legs syndrome (RLS). The questionnaires were distributed after informed consent.

The MISSOULA-Vitas Quality of Life Index-15 (MVQOLI-15) questionnaire was distributed during the initial phase of this study. The abbreviated version of the original MVQOLI-25 consists of 15 items adapted from the English version. It assesses five key dimensions, including symptoms, functional ability, interpersonal relationships, well-being, and spirituality, as well as an overall quality of life score. Scores range from -30 to 30, with higher values reflecting fewer symptoms, greater functional capacity, stronger interpersonal connections, and enhanced well-being and spirituality.

The Fatigue Severity Scale (FSS) was also administered, using the Greek version adapted and standardized by Z. Katsarou and S. Bostantopoulou [18]. The FSS evaluates the intensity of fatigue and helps distinguish it from clinical depression. Participants rated their level of fatigue through nine questions, using a scale from 1 (strongly disagree) to 7 (strongly agree).

Finally, a questionnaire was distributed based on diagnostic criteria for restless legs syndrome (RLS), adapted and standardized by the Muscle and Metabolism Study Unit [19]. For RLS, we implemented standard diagnostic methodology, i.e., the essential criteria of the International RLS Study Group. More specifically, RLS was confirmed in all patients who fulfilled all four criteria.

Data for continuous variables are given as a mean value and standard deviation (SD), whereas categorical variables are presented as the number of cases (*n*) and percentage (%). We used the χ^2^ test for categorical variables and the Kruskal–Wallis *H* test for continuous variables. Kaplan–Meier survival curves were generated for survival probability and analyzed by the log-rank (Mantel–Cox) test. Statistical analyses were performed using the Statistical Package for Social Sciences 29.0 (SPSS 29.0) (SPSS Inc., Chicago, IL, USA). *p*-Values ≤ 0.05 (two-sided) were regarded as statistically significant. Regarding missing data handling, pairwise deletion was applied. Because this study included every eligible patient, sample size calculation was not performed.

## 3. Results

**Figure 1:**
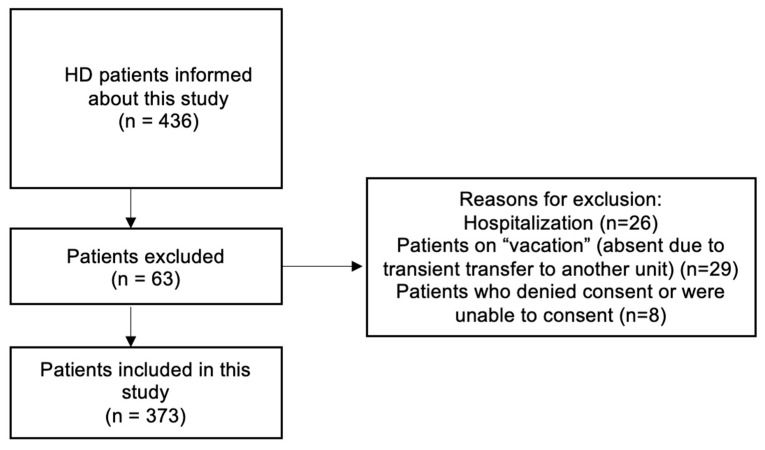
Flow diagram of the patients’ enrollment.

Among the 436 HD patients who were informed about this study, 373 were included, resulting in a response rate of 85.6% (Figure 1). Overall, the survey included 373 patients (125 females; mean age 65.7 ± 14.8 years) with ESRD treated with chronic HD. The clinical profile of the sample is shown in Table 1.

Between the different types of vascular access, there were significant differences regarding the age (*p* < 0.001) and gender (*p* = 0.011) of the patients, the FSS score (*p* = 0.041), and three dimensions of the MISSOULA scale, including symptoms (*p* = 0.011), interpersonal relationships (*p* < 0.001), and spirituality (*p* = 0.005), as well as the overall quality of life (QoL) (*p* = 0.008) (Table 2). The MISSOULA and FSS raw scores are presented in Supplemental Tables S1 and S2.

**Figure 2:**
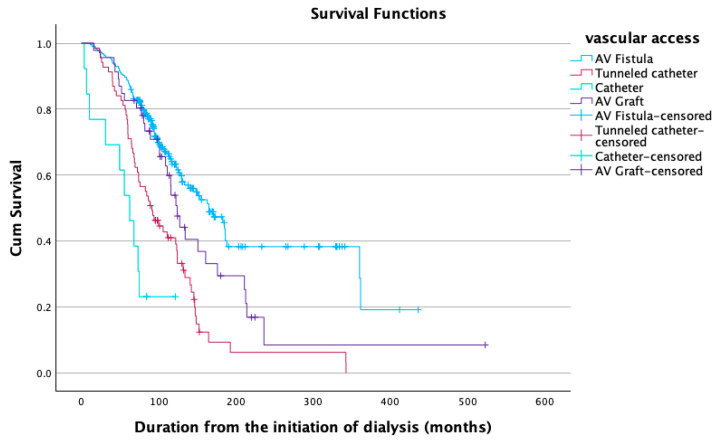
Survival analysis regarding the type of vascular access.

The patients were categorized based on the type of vascular access: fistula (*n* = 227), tunneled catheter (*n* = 71), nontunneled catheter (*n* = 14), and AV graft (*n* = 46). A Kaplan–Meier survival analysis [20] was conducted to compare the four different types of vascular access in terms of survival (Figure 2). A different proportion of censored cases was observed among the various types. The mean duration of follow-up was 113.5 ± 71.9 months. The participants with a fistula had a median survival time of approximately 165 months (95% CI, 17 to 132 months). This survival duration was longer than that of the other types of vascular access. A log-rank test was performed to determine whether there were differences in the survival distributions among the different types of vascular access. The survival distributions for the four types of vascular access were statistically significantly different, i.e., χ^2^ =53.038, *p* < 0.001. Pairwise comparisons were conducted to identify which types of vascular access had significantly different survival distributions. All pairwise comparisons were significant except AV fistula versus AV graft.

A Cox regression analysis was also conducted, which showed that both age (*p* < 0.001) and the type of vascular access (*p* < 0.001) were statistically significant. More specifically, in the multivariable Cox proportional-hazards model, age emerged as a consistent, independent risk factor. Every additional year of age increased the instantaneous hazard of the event by about 5% (HR = 1.049, 95% CI 1.036–1.063, *p* < 0.001). Clinically, this means that a patient who is ten years older is approximately 50% more likely to experience the event at any given time than a younger counterpart of similar clinical profile. Vascular access type was also a strong predictor of risk. Compared with the reference category of arteriovenous fistula, tunneled catheters nearly doubled the hazard (HR = 1.96, 95% CI 1.39–2.77, *p* < 0.001) and nontunneled catheters carried more than a four-fold increase in hazard (HR = 4.31, 95% CI 2.13–8.70, *p* < 0.001) constituting the riskiest option, whereas AV grafts showed no statistically significant difference from fistula.

## 4. Discussion

Despite the rapid advancement of technology and the knowledge that has been acquired in the field of hemodialysis, advancements in vascular access have unfortunately not kept pace with the rapid development seen in other areas of hemodialysis. Vascular access remains the weak link of the method even today. Significant steps have been taken in the evolution of and improvement in vascular access; however, they have not yet met the expectations of the scientific community. The addition of scientific knowledge on the subject is deemed essential. Therefore, the present study aims to contribute to the scientific knowledge and examine the correlation between the type of vascular access and patient fatigue, RLS, the assessment of early or delayed referral to a nephrologist, and, ultimately, the impact on the quality of life of hemodialysis patients, which is the primary goal of implementing hemodialysis with an artificial kidney. It is noteworthy to mention that this is the first time this issue has been investigated in an epidemiological survey in Greece.

Based on the findings of the present study, fistula represents the most common type of vascular access. This finding is in accordance with the literature [21]. Regarding demographic data, age and gender are statistically significantly different in patients with different types of vascular access. The younger patients in this study had fistulas, whereas the older patients had tunneled catheters. Fistulas are also more common in males than females [22,23]. The assessment by a nephrologist did not differ significantly based on the type of vascular access.

It is noteworthy to mention that survival was higher in those patients with fistulas, i.e., 165 months (95% CI, 17 to 132 months). This finding is in accordance with many lines of evidence [22,23,24,25,26,27,28,29,30,31,32,33,34,35]. More specifically, the present study revealed higher survival rates in the group of patients with a fistula, followed by the group of patients with a graft, and then by those with a permanent catheter, while the lowest survival was observed in the group of patients with a temporary catheter. Thus, the present study showed that AV fistulas carry the lowest risk, whereas nontunneled catheters appeared particularly hazardous and should be avoided when alternative types of vascular access are feasible.

Patients with end-stage renal disease (ESRD) undergoing hemodialysis also experience a significant symptom burden both during and after treatment, which negatively affects their quality of life [36]. Fatigue is one of the most common and debilitating symptoms experienced by patients, involving physical, psychological, and emotional dimensions [36,37,38]. In the present study, Fatigue Severity Scale (FSS) scores varied significantly based on the type of vascular access. The patients with tunneled catheters had the highest fatigue levels, while those with arteriovenous fistulas had the lowest scores.

Beyond survival, quality of life should be taken into consideration in determining the most appropriate type of vascular access for hemodialysis patients. Individuals with end-stage renal disease (ESRD) often experience reduced health-related quality of life and higher levels of depression, which may impact overall survival. Among the contributing factors, vascular access type stands out as a potentially modifiable factor influencing quality of life outcomes [39,40,41]. Regarding the MISSOULA-Vitas Quality of Life Index-15 (MVQOLI-15) questionnaire, the dimensions that differ significantly according to the various types of vascular access are symptoms, interpersonal relationships, spirituality, and overall QoL. It is noteworthy to mention that the MVQOLI-15 was chosen because it is tailored to assess multidimensional quality of life in patients with advanced illness and includes domains particularly relevant to our study population, offering a nuanced evaluation in the context of chronic and progressive conditions.

This paper concludes by suggesting early nephrology referrals to promote AVF creation, although the results did not show differences in nephrology referral across different access types. However, the *p*-value was marginal, and in early referral, the frequency of AVF was significantly higher. The aforementioned outcome might be influenced by delays in decision-making or medical contraindications among patients referred early and could have led to catheter use despite timely referral.

Regarding the limitations of the present study, the cross-sectional design for quality-of-life parameters prevents the identification of causal associations, although it suits the descriptive goals of the present study. In addition, the Attica region, where this study was conducted, restricts generalizability. Thus, future studies including a greater sample size across Greece are needed for broader Greek representation. Also, the lack of sample-size calculation should be considered.

## 5. Conclusions

The current study covers the epidemiology of vascular access in chronic hemodialysis patients in Greece and the impact of vascular access type on fatigue, survival, and quality of life. It indicates the lack of appropriate access that necessitates improvement in planning, as well as earlier educational consultations with nephrologists to lower catheter dependency and enhance the prospects of prolonged survival post-dialysis. There is a need for a national policy in Greece aimed at improving the management of catheter complications, enhancing the use of fistulas, and investigating the psychosocial repercussions of the vascular access interface on patients undertaking dialysis. Whenever feasible, AV fistulas remain the safest option. Catheters—especially nontunneled ones—should be used only when unavoidable and replaced promptly. AV grafts did not differ from fistulas in this cohort, but the estimate was imprecise; thus, further study may be warranted.

### Future Directions

Future research should concentrate on enhancing early nephrology referrals to guarantee timely AVF creation and decrease central venous catheter use. The improvement in outcomes requires studying barriers to AVF placement and developing new technologies, including bioengineered grafts and endovascular AVF creation. Research on fatigue and quality of life needs to be extended to include studies on interventions that decrease fatigue over the long term. The development of global policies and surveillance programs must occur to decrease catheter dependency and enhance care quality, which will lead to better patient survival and improved quality of life. Relevant catheter-related complication data (e.g., infection and thrombosis rates) should be considered. In addition, staff training programs, improved referral pathways for early AVF creation, and enhanced patient education on vascular access options could constitute practical policy implications. Future directions, such as investigating barriers to AVF use and evaluating the impact of early referral programs, will result in improved vascular access management and enhanced survival rates and quality of life for patients undergoing hemodialysis.

## Figures and Tables

**Table 1:** Clinical profile and type of vascular access of ESRD patients (N = 373).

		Ν	%
Gender	Male	242	65.9
	Female	125	34.1
Age, mean (SD)		65.7 (14.8)	
Primary kidney disease	Diabetic nephropathy	69	19.9
	Glomerulonephritis	58	16.8
	Vascular kidney disease	5	1.4
	Hypertension	26	7.5
	Polycystic kidney disease	34	9.8
	Pyelonephritis	20	5.8
	Interstitial nephritis	4	1.2
	Unknown etiology	108	31.2
	Other causes	22	6.4
Type of vascular access	Fistula	227	63.4
	Tunneled catheter	71	19.8
	Nontunneled catheter	14	3.9
	AV graft	46	12.8

**Table 2:** Differences in the main demographic and fatigue- and quality-of-life-related parameters between types of vascular access.

Type of Vascular Access	Fistula	Tunneled Catheter	Nontunneled Catheter	AV Graft	*p*-Value
**Age** (mean ± SD)	63.54 ± 14.36	71.20 ± 13.45	66.08 ± 21.09	68.98 ± 13.69	<0.001
**Gender** Male [*n* (%)]	161 (72.2%)	40 (57.1%)	10 (71.4%)	24 51.1%)	0.011
**Assessment by nephrologist** Yes [*n* (%)]	153 (70.5%)	36 (53.7%)	10 (76.9%)	27 (61.4%)	0.055
**RLS** Yes [*n* (%)]	39 (18.5%)	18 (28.6%)	0 (0%)	9 (23.1%)	0.108
**FSS** (mean ± SD)	4.15 ± 1.65	4.79 ± 1.25	4.37 ± 2.02	4.40 ± 1.64	0.048
**Overall QoL** (mean ± SD)	3.56 ± 0.89	3.26 ± 0.68	2.92 ± 1.19	3.37 ± 0.93	0.005

## Data Availability

The original contributions presented in this study are included in this article. Further inquiries can be directed to the corresponding author.

## Supplementary Materials

The following supporting information can be downloaded at https://www.mdpi.com/article/10.3390/jcm14134571/s1: Table S1. MISSOULA raw scores, Table S2. FSS raw scores.

## Abbreviations

The following abbreviations are used in this manuscript:

VA	vascular access
ESRD	end-stage renal disease
HD	hemodialysis
*AVF*	arteriovenous fistula
*AVG*	arteriovenous graft
*CVCs*	central venous catheters
*CKD*	chronic kidney disease
*TDCs*	tunneled dialysis catheters
*USRDS*	United States Renal Data System
*LICs*	low-income countries
*HICs*	high-income countries
*PD*	peritoneal dialysis
*EDUs*	Extracorporeal Dialysis Units
*CHUs*	Chronic Hemodialysis Units
*QoL*	quality of life
*MVQOLI-15*	Missoula-Vitas Quality of Life Index-15
*FSS*	Fatigue Severity Scale
*RLS*	restless legs syndrome
