# Supplementary material for: Epidemiology of Vascular Access in Patients Undergoing Chronic Hemodialysis Treatment in Greece
Source: J Clin Med. 2025 Jun 27;14(13):4571. doi: 10.3390/jcm14134571 (PMC12249874; doi:10.3390/jcm14134571)
Supplement: Supplementary file 1 [file jcm-14-04571-s001.zip › jcm-3675412-supplementary.pdf]

Table S1: MISSOULA raw scores.

Overall QoL	Q1	Q2	Q3	Q4	Q5	Q6	Q7	Q8	Q9	Q10	Q11	Q12	Q13	Q14	Q15
3	0	3	4	-1	3	4	1	3	5	-1					
3	0	4	3	0	3	4	1	3	5		-3	4	2	4	
3	0	3	5	-2	4	5	1	-3	5	-1	-4	5	2	3	5
3	-1	3	4	-2	4	4	1	3	4	1	-4	4	-1	3	4
3	0	0	3	1	3	5	1	4	5	0	-4	4	-1	4	5
3	0	3	3	-2	-3	4	2	3	3	0	4	5	-2	0	4
3	0	3	4	-1	0	4	1	3	4	0	-3	4	1	3	4
3	0	4	5			5	2	4	5	2		5	2	4	5
3	0	3	4	-1	3	4	2	3	3	0	-3	3	-2	0	4
3	2	4	4	-2	4	5	2	4	5	-1	4	5	2	4	5
5	2	4	1	0	4	5	2	4	5	2	0	5	0	4	5
4	0	3	4	-1	3	4	1	0	4	-1	-4	5	-1	3	4
4	1	3	2	1	3	4	1	3	4	1	3	4	1	4	4
4	1	3	2	1	3	4	1	3	4	1	3	4	1	4	4
3	1	3	4	0	3	4	1	3	4	1	0	4	0	3	4
4	2	3	4	-1	3	4	1	3	4	1	-3	4	-1	3	4
2	-1	-3	4	-1	0	4	1	3	4	1	-4	4	-2	0	3
3	-1	3	2	-1	3	4	0	3	4	0	-3	4	-1	3	4
4	1	4	4	-1	4	4	2	4	4	1	-3	4	1	3	4
4	2	4	1	1	0	4	2	4	5	1	-4	5	0	4	5
3	0	3	5	-2	3	3	1	0	4	1	-3	3	1	4	5
4	2	3	2	1	-3	4	1	3	5	2	-3	5	-2	4	5

3	1	3	2	0	3	4	1	3	5	1	-3	4	-1	3	4
3	1	3	2	-1	3	4	1	0	4	1	-4	4	-2	3	4
3	1	3	4	-2	3	4	1	0	3	1	-4	4	-1	3	4
3	1	3	2	1	-3	4	1	3	4	2	-3	4	-1	4	4
3	-1	-3	4	-1	3	4	1	0	4	-1	-4	4	-2	3	4
4	1	3	3	-2	3	4	1	3	4	1	-4	4	-1	4	4
2	0	0	3	-1	0	4	0	0	3	-1	-4	4	-2	0	4
4	1	3	4	-1	3	4	1	3	4	-1	-3	2	-1	-3	4
3	1	3	4	1	3	4	1	3	3	0	-3	4	1	3	4
2	-1	3	4	1	3	4	-1	-3	4	-1	-3	4	-1	0	3
2	0	-3	4	-1	0	4	0	0	3	1	-3	3	-2	0	3
3	1	3	3	0	0	4	1	3	4	0	0	4	0	3	4
4	0	3	4	-1	3	5	1	3	4	-1	3	4	-1	0	4
4	2	0	2	-1	-3	4	1	0	5	0	-4	5	-1	4	5
4	1	3	2	1	-3	4	1	3	5	1	-3	4	1	4	5
4	0	3	4	-1	3	4	0	0	2	-1	-4	4	-1	3	4
3	0	-3	1	1	3	2	1	3	4	1	-3	4	1	3	4
5	2	3	1	2	-4	5	2	4	5	1	3	4	2	4	5
5	1	4	2	1	-3	5	2	3	4	1					
	1	3	3	0	0	5	1	3	4	0	3	4	1	3	4
3	-2	4	3	-2	4	4	1	0	5	-2	4	4	-1	-3	5
3	0	3	2	-1	4	4	1	3	5	1	0	4	2	3	4
5	2	4	1	2	0		2	0	5	0	4	5	0	4	5
4	1	0	4	-1	0	5	1	3	4	1	-3	4	0	3	4
3	1	0	2	2	0	4	1	3	4	0	4	4	0	3	4

4	2	3	1	1	-3	1	2	4	5	-1	3	5	2	4	5
4	2	3	2	1	0	5	1	3	5	0	0	5	0	4	5
3	0	4	4	0	0	4	1	3	4	1	-4	5	-1	-3	5
3	0	3	3	1	0	4	0	3	3	0	-3	3	1	-3	3
3	0	-3	4	-1	3	4	0	0	4	1	-4	4	0	0	3
3	1	-3	4	-1	3	5	-2	3	5	-2	3	5	2	4	5
2	0	0	4	-1	-3	4	2	0	3	1	-4	4	-2	-3	4
2	-1	3	5	-2	4	3	1	3	5	2	0	4	0	-3	4
5	2	-4	1	2	-4	5	2	4	5	2	3	4	2	4	5
2	-1	-3	5	-2	4	4	2	0	4	-2	-4	5	-2	0	5
4	1	-3	2	0	0	5	1	0	4	1	-4	4	0	0	4
3		0	4	-1	3	3	0	0	3	0	-3	4	0	-3	4
4	1	-3	3	1	3	4	1	3	4	-1	-3	4	-1	0	3
4	1	4	2	-1	3	4	2	4	5	-1	-3	5	-1	4	5
3	1	3	5	-1	3	4	2	3	4		4	4	1	3	4
	1	3	4	1	-3	4	1	3	4	-2	-3	4	-1	4	5
4	1	3	2	-1	-3	5	1	3	4	1	-4	5	-2	3	5
4	1	3	3	1	0	5	2	4	5	-1	-3	5	-2	3	5
4	2	3	1	1	3	4	2	4	5	1	0	5	0	4	5
4	1	0	2	-1	3	4	1	3	4	-1	-3	4	0	3	4
				1	-4	5	1	3	4		0	4	1	3	4
3	2	3	2	-1	3	4	1	3	4	-1	-3	4	-1	3	4
											0	5	0	3	5
3	0	3	2	-1	3	4	1	3	4	1	3	4	1	3	4
1	0	3	5	-2	4	5	-1	-3	4	1	-4	5	-2	0	4

4	2	3	4	-1	4	5	1	4	5	0	0	5	2	4	5
3	0	3	3	1	3	4	0	-3	3	1	-3	4	-1	3	4
3	0	3	4	-1	3	4	-1	-3	2	-1	-3	4	0	0	3
5	2	4	1	2	-4	5	2	4	5	2	3	5	1	4	5
4	1	3	2	1	-3	4	1	3	4	1	3	4	1	3	4
4	1	3	2	1	-3	4	1	3	4	1	3	4	1	3	4
4	1	3	3	1	-3	4	0	3	3	1	0	4	0	3	4
4	1	3	2	1	-3	4	1	3	4	1	0	4	0	3	4
3	0	3	3	-1	-3	4	1	0	3	1	0	4	0	3	4
3	0	3	4	-1	3	3	0	-3	3	1	-3	3	-1	-3	3
3	0	0	3	-1	3	2	-1	0	3	1	-3	3	-1	-3	3
4	1	3	2	1	-3	4	2	4	4	1	3	4	1	3	4
4	1	3	4	-1	3	4	1	3	4	1	0	4	0	3	4
2	-1	3	4	-1	3	3	0	-3	3	1	-3	3	-1	0	3
4	1	3	2	1	-3	4	1	3	4	1	0	4	0	3	4
3	0	3	4	-1	3	4	-1	-3	3	1	-3	4	-1	0	4
5	2	4	1	2	-4	5	2	4	5	2	0	5	0	3	4
3	-1	0	4	-1	3	2	-1	-3	3	1	-3	4	-1	-3	4
5	2	4	1	2	4	5	1	3	4	1	3	4	1	3	4
3	-1	0	4	-1	3	3	-1	-3	2	0	-3	3	-1	-3	3
5	1	3	1	2	-4	5	2	3	5	2	3	4	1	4	4
3	0	3	4	-1	3	3	0	0	3	1	-3	4	0	3	3
3	1	3	3	1	0	3	1	0	3	1	0	4	0	3	4
4	1	3	2	2	-4	5	2	4	5	2	3	5	1	4	5
4	1	3	2	1	3	4	1	3	4	1	0	4	0	0	3

4	1	3	2	1	-3	4	1	3	4	1	3	4	1	3	4
4	1	3	2	1	-3	4	1	3	4	1	3	4	1	3	4
4	1	3	2	0	-3	4	1	0	3	1	0	4	0	3	4
4	1	3	3	1	0	5	1	3	5	1	0	5	0	4	5
4	1	3	2	1	3	5	2	3	5	-1	-3	4	0	4	5
1	-2	3	5	-2	4	4	1	-3	2	-1	-4	4	1	-3	2
4	1	3	2	1	-3	4	1	3	4	1	0	4	0	3	4
5	1	4	5	1	-3	5	2	3	5	2	0	5	0	4	5
4	2	-4	1	1	3	4	2	3	4	1	-3	4	0	3	4
4	0	0	2	2	-3	4	2	4	4	0	3	5	2	4	4
4	1	3	3	1	-3	5	1	3	3	1	-3	4	-1	3	3
4	1	3	2	1	-3	4	1	3	4	1	-3	4	-1	3	4
3	-1	0	4	-1	0	4	1	0	4	-1	-3	4	-1	-3	4
4	1	3	2	1	-3	4	1	0	3	1	0	4	0	3	4
3	-1	3	4	-1	3	4	0	0	3	1	-3	4	-1	0	4
4	1	3	3	0	3	4	1	3	3	1	0	4	-1	3	4
3	1	3	3	0	-3	4	1	3	4	1	3	4	1	3	4
4	1	3	2	1	-3	4	1	3	4	1	0	4	0	3	4
4	1	3	2	1	-3	4	1	3	4	1	-3	4	-1	3	4
5	2	4	1	2	-4	5	2	4	5	2	0	5	0	4	5
2	-1	3	4	-2	4	4	-1	-3	2	1	-3	4	-1	-3	4
4	1	4	1	1	-3	4	1	3	4	1	3	4	0	3	4
3	1	3	2	-1	3	4	0	0	3	1	-3	4	-1	0	4
4	1	3	2	1	-3	4	1	3	4	1	3	4	0	3	4
4	-1	3	4	-1	3	3	-1	-3	2	1	-3	3	-1	-3	3

4	1	3	2	1	-3	4	1	3	4	1	3	4	1	3	4
4	1	3	2	1	-3	4	1	3	4	1	3	4	1	3	4
3	-1	3	4	-1	3	4	-1	-3	3	1	-3	3	-1	-3	4
4	1	3	3	0	-3	4	1	3	3	1	0	4	0	3	4
	0	3	3	-1	3	4	1	0	3	1	3	4	1	3	4
4	1	3	4	-1	-3	4	1	0	3	1	3	4	0	3	4
3	1	3	3	-1	0	4	1	3	3	1	0	4	0	3	4
4	1	3	2	1	-3	4	1	0	4	1	0	4	0	3	4
4	1	3	2	1	-3	4	0	0	3	1	0	4	0	3	4
4	1	3	2	-1	-3	4	1	3	3	1	3	4	0	3	4
3	0	3	3	-1	-3	4	1	3	3	1	0	4	0	3	4
4	1	3	2	-1	0	4	1	3	3	1	0	4	1	3	4
4	1	3	4	-1	3	4	0	0	3	1	0	4	0	3	4
3	1	3	4	-1	3	4	0	0	3	1	3	4	0	3	4
4	1	3	2	1	-3	4	1	3	4	1	3	4	1	3	4
4	1	0	3	-1	-3	4	0	0	3	1	0	4	0	3	4
5	2	3	1	2	-3	4	1	3	4	-1		3	2	4	4
5	2	4	1	2	-4	5	2	4	5	2	0	5	0	4	5
3	0	3	4	-1	3	3	0	0	3	1	0	4	-1	0	3
3	1	3	3	0	0	3	-1	0	3	1	3	4	0	3	4
5	2	3	1	2	-4	5	2	4	5	2	0	5	0	4	5
4	1	3	2	1	-3	4	1	3	4	1	0	4	0	3	4
4	1	3	3	0	-3	4	1	3	3	1	0	4	-1	0	4
4	1	3	2	1	-3	4	1	3	4	1	-3	4	-1	3	4
2	-1	3	4	1	3	3	0	-3	3	1	3	3	1	0	3

3	1	3	3	-1	3	4	0	3	3	1	-3	3	-1	3	4
4	1	3	2	1	0	4	1	3	4	1	3	4	1	3	4
3	-1	3	4	-1	3	4	0	0	3	1	-3	4	0	0	4
4	1	3	3	-1	3	4	1	0	3	1	0	4	-1	3	4
4	1	0	4	1	3	4	0	0	3	0	-3	3	-1	3	3
4	1	3	2	1	-3	4	1	3	4	1	3	4	0	3	4
3	0	3	2	1	-3	4	1	3	4	1	0	4	-1	3	4
4	1	3	2	1	-3	4	1	3	4	1	-3	4	-1	3	4
3	0	3	3	-1	3	4	0	-3	2	1	-3	4	-1	0	3
4	1	3	2	1	-3	4	1	3	4	1	3	4	1	3	4
4	1	3	2	1	-3	4	1	3	4	1	3	4	1	3	5
2	-1	0	4	-1	3	4	1	3	3	-1	-3	3	-1	0	3
3	-1	3	4	-1	3	4	0	0	3	-1	-3	4	-1	-3	4
5	1	3	2	1	3	4	1	3	4	1	3	4	2	3	4
4	1	4	2	-1	3	4	1	3	4	-1	-3	4	-1	3	4
4	1	3	2								0	4	0	0	4
4	1	3	2	0	4	5	2	4	5	1	0	5	0	4	5
4	1	3	2	1	-3	4	1	3	4	1	-3	5	-1	-3	4
3	1	3	2	-1	3	4	1	3	4	1	0	4	-1	3	4
4	0	0	2	1	3	4	1	3	4	1	3	4	1	4	4
3	1	3	3	-1	0	4	1	0	4	1	3	4	1	3	4
4	-1	3	4	-1	3	4	1	3	4	0	-3	4	-1	-3	3
2	-1	3	4	-1	3	4	1	3	3	1	-3	4	-1	-3	4
4	1	3	4	-1	3	4	1	-3	3	-1	-3	4	-1	0	4
4	1	3	4	-1	3	4	0	0	4	1	0	4	-1	0	4

5	2	3	1	2	-4	5	2	4	5	-2	4	5	2	4	5
2	0	3	3	-1	3	4	1	3	3	-1	-3	4	-1	-3	4
3	1	3	2	1	-3	4	1	3	4	1	3	4	1	4	4
4	1	3	2	1	3	3	0	0	3	1	3	4	0	3	4
4	1	3	2	2	-4	4	1	3	4	1	0	4	0	3	4
5	2	-4	1	2	-4	5	2	4	5	2	-4	5	0	4	5
3	1	3	4	-1	3	4	1	0	4	0	-3	4	0	0	3
3	0	0	3	-1	3	3	0	0	3	-1	-3	3	-1	-3	4
5	2	4	1	2	-4	5	2	4	5	1	0	5	0	4	5
4	1	3	3	-1	3	4	1	3	4	1	3	4	1	3	4
3	0	3	3	-1	3	4	1	3	4	1	0	4	-1	3	4
3	-1	3	4	-1	3	4	-1	-3	3	-1	0	4	0	0	3
1	-1	3	4	-1	3	3	-1	-3	3	-1	-3	3	-1	-3	4
3	0	3	4	1	-3	4	1	0	4	1	0	3	-1	0	4
3	0	3	4	-1	3	4	1	0	4	0	-3	4	-1	0	4
3	-1	3	4	1	0	4	1	3	4	1	3	4	-1	-3	4
4	0	0	3	-1	-3	4	1	0	3	1	-3	4	-1	-3	4
5	1	3	2	1	-3	4	1	3	4	1	3	4	1	3	4
4	1	3	2	-1	3	4	1	3	4	-1	3	4	1	3	4
3	2	4	2	1	3	3	-1	3	4	-1	-3	4	1	3	4
4	1	0	3	0	0	4	1	3	4	1					
4	1	3	2	1	0	3	1	0	3	1	-3	4	-1	3	4
4	1	0	2	-1	3	4	1	0	3	1	0	4	0	3	4
2	-1	0	4	-1	3	3	-1	-3	2	1	0	3	-1	-3	3
3	0	0	3	-1	3	4	0	0	3	1	0	4	1	3	4

2	-1	0	4	-1	3	3	1	3	4	0	3	3	-1	-3	2
4	1	3	2	1	-3	4	1	3	4	1	3	4	1	3	4
4	1	3	2	1	-3	4	1	3	4	-1	0	4	0	3	4
3	0	0	3	-1	3	4	0	0	3	1	-3	4	-1	3	3
5	2	3	1	2	-3	5	2	4	5	2	3	5	1	4	5
4	0	3	3	-1	-3	4	1	3	4	1	3	4	1	3	4
3	0	3	4	-1	3	3	0	0	2	1	-3	4	-1	0	3
3	1	3	2	0	3	3	1	3	4	-1	3	4	-1	0	4
3	0	3	4	-1	3	4	-1	-3	3	0	-3	3	-1	-3	4
3	2	3	2	2	3	4	1	-3	4	0	-3	4	0	3	4
4	1	3	2	1	-3	4	1	3	4	1	3	4	1	3	4
4	2	3	1	2	-4	5	2	4	5	0	0	5	0	4	5
4	1	3	2	1	-3	4	1	3	4	1	-3	4	1	3	4
5	2	3	1	2	-4	5	2	4	5	2	4	5	2	4	5
4	1	3	2	1	0	4	0	0	3	1	0	3	0	3	4
5	2	3	1	2	-4	5	2	4	5	2	4	5	1	4	5
4	1	3	2	-2	4	4	1	3	4	-1	0	4	0	0	3
2	-1	0	4	-1	3	4	1	-3	3	1	-3	3	-1	-3	3
4	1	3	2	-1	3	3	1	3	4	1	-3	4	-1	0	3
3	1	3	4	1	-3	4	1	3	4	1	-3	4	-1	3	4
2	-1	3	4	-1	3	4	-1	-3	3	1	-3	4	-1	-3	3
2	-1	3	4	0	3	4	0	0	4	-1	-3	3	0	0	3
5	2	4	1	2	-3	5	2	4	5	0	4	5	2	4	5
3	1	3	2	1	0	4	1	3	4	1	3	4	1	3	4
2	-1	0	4	-1	3	4	1	0	3	1					

2	-2	0	5	-2	4	4	2	4	5	-1	-4	1	-2	4	1
2	-1	3	4	-1	3	4	1	3	4	-1	-3	3	1	0	4
3	0	0	4	-1	3	3	1	0	3	1	-3	3	-1	-3	3
1	-2	4	5	-2	4	5	0	0	3	-2	-4	1	-2	4	3
4	1	3	2	1	-3	4	1	3	4	1	-3	4	-1	0	4
4	1	3	2	1	-3	4	1	3	4	1	3	4	1	3	4
3	0	0	3	0	0	3	0	0	3	0	0	3	0	0	3
4	1	3	2	1	-3	4	1	3	4	1	0	4	0	3	4
4	1	0	2	1	3	4	1	3	5	-1	-3	4	-1	3	5
3	0	3	3	-1	3	5	2	4		-1	3	5	2	4	5
				1	0	4	1	3	4	1	-3	4	-1	3	4
3	0	0	3	0	0	3	0	0	3	0	0	3	0	3	3
4	1	0	1	2	-3	5	2	3	4	2	-4	5	-1	4	5
4	1	3	2	1	0	4	1	3	4	1	0	4	-1	3	4
4		3	2	1	-3	4	1	3	4	1	0	4	1	3	4
4	1	3	3	1	-3	3	1	3	4	0	0	3	0	0	3
4	1	3	2	1	-3	4	1	3	4	1	0	4	0	3	4
2	-1	3	4	-1	3	3	-1	-3	3	-1	-3	3	-1	-3	4
3	-1	0	4	-1	3	3	0	-3	3	-1	-3	3	-1	-3	3
2	-1	0	4	-1	3	3	-1	-3	2	-1	0	3	-1	-3	3
5	1	3	2	2	-3	4	1	3	4	1	3	4	1	3	4
3	1	0	4	-1	3	4	1	3	3	0	0	4	0	0	3
4	1	3	2	1	-3	4	1	3	4	1	0	4	1	3	4

4	1	3	2	1	0	4	1	3	4	1	0	4	0	3	4
3	0	3	3	-1	3	3	0	-3	3	1	-3	3	-1	0	3
4	0	3	3	-1	3	3	1	0	3	1	-3	4	-1	3	4
4	1	3	2	1	-3	4	1	0	4	0	0	4	0	3	4
4	1	3	2	1	3	4	1	3	4	0	0	4	0	3	4
2	-1	3	4	-1	3	4	0	0	3	-1	-3	4	-1	-3	4
2	-1	3	4								-3	3	-1	-3	4
1	1	0	2	-1	3	4	0	0	4	0	0	3	-1	0	4
2	1	0	4	1	-3	2	1	3	4	-1	0	4	0	0	3
5	1	3	2	1	-3	4	1	3	4	1	0	4	0	3	4
4	1	3	4	1	3	4	1	3	4	-1	0	3	0	0	3
2	-1	3	4	-1	3	3	1	0	3	-1	3	3			
3	0	0	3	1	0	3	0	0	3	0	0	3	0	0	3
4	2	3	2	-1	3	3	-1	0	3	-1	-3	4	-1	0	3
1	-1	3	4	-1	3	4	1	3	4	-1	-3	3	-1	-3	4
3	1	3	4	-1	3	3	1	3	4	1	-3	4	-1	0	4
2	-1	0	4	-2	4	4	0	0	3	-1	-4	3	-2	-4	4
4	1	3	4	-1	3	4	1	3	4	1	3	4	0	3	4
4	0	0	3	-2	4	5	2	3	4	-2	3	5	0	0	4
4	1	0	2	1	-3	3	1	3	3	1	0	4	0	3	4
3	0	0	4	-1	3	4	1	3	4	-1	-3	4	-1	0	3
3	0	3	4	-1	3	4	1	0	3	-1	-3	4	-1	-3	4
4	1	3	3	-1	3	3	1	3	4	0	0	4	-1	4	5
4	1	3	2	-1	3	5	1	4	4	0	0	5	-1	0	5

5	1	3	2	1	-3	4	1	3	4	1	0	4	0	3	4
4	1	3	2	1	-3	4	1	3	4	1	3	4	1	3	4
4	1	3	4	1	-3	5	2	3	3	-1	3	5	-1	3	4
5	2	4	1	2	-4	5	2	4	5	-1	0	5	0	4	5
4	1	3	3	1	0	4	1	3	3	0	3	4		3	5
4	2	3	2	1	-3	5	-1	3	5	-1	3	5	1	4	5
4	1	3	2	1	-3	5	-1	3	5	-2	3	5	2	4	5
4	1	0	4	-1	3	4	0	-3	3	-1	-3	4	-1	0	4
2	1	3	3	-1	-3	4	1	3	4	1	0	4	1	3	4
3	0	3	4	-1	3	4	1	0	4	0	0	3	-1	-3	4
2	-1	3	4	-1	3	4	0	-3	3	0	-3	4	-1	-3	3
3	1	3	3	-1	3	4	1	0	4	0	-3	3	-1	-3	3
3	1	0	3	-1	3	3	1	0	3	1	-3	3	-1	-3	3
3	1	3	4	-1	0	4	1	3	4	-1	-3	4	-1	3	4
4	1	3	2	0	3	4	1	3	5	-1	3	4	1	3	4
3	-1	0	5	-1	4	4	1	-3	4		-3	4	-1	-3	4
2	-1	0	4	-1	3	3	1	0	3	1	-3	3	-1	-3	4
2	-1	0	4	-1	3	4	0	0	3	1	-3	4	-1	-3	3
3	0	3	4	-1	3	3	1	0	3	1	-3	3	-1	0	4
4	2	4	1	2	-4	5	2	4	5	2	4	5	2	4	5
4	1	3	2										1	3	4
3	0	3	4	1	3	4	1	3	4	1	-3	4	-1	-3	4
3	0	3	4	-1	3	3	1	0	3	1	-3	4	-1	0	4
4	1	3	2	1	-3	4	1	3	4	1	0	4	0	3	4

3	1	3	3	-1	3	4	0	0	3	-1	0	4	-1	3	4
2	0		4	-1	3	3	1	-3	3	0	-3	3		0	3
4	1	3	2	1	0	4	2	4	5	-1	-3	4	2	3	4
2	-1	3	4	-2	3	5	0	-3	2	0	-4	3	-2	-4	4
4	2	4	4	0	3	5	2	4	5	2	0	5	2	4	5
3	1	3	4	-1	-3	4	-1	-3	4	-1	0	4	0	0	4
3	0	3	4	-1	3	4	0	0	3	1	-3	4	-1	0	3
4	-1	3	4	-1	-3	4	0	0	3	0	3	3	0	0	3
3	0	3	4	-1	3	4	0	-3	3	0	-3	4	-1	-3	4
4	0	0	2	0	3	4	1	3	5	-1	0	5	1	4	4
4	1	3	2	1	3	5	1	0	5	0	3	5	2	4	5
4	1	0	2	1	3	4	1	0	4	-1	-3	4	-1	3	4
4	0	0	2	1	3	4	2	4	5	-1	-4	5	2	3	5
4	-1	3	4	-1	3	4	2	3	4	-1	-3	3	-1	0	4
4	1	3	2	1	-3	4	1	3	4	1	4	5	1	4	4
3	1	3	3	-1	3	4	1	3	4	-1	-3	4	-1	3	4
2	-1	-3	4	-1	3	3	0	0	3	0	-3	4	-1	-3	3
4	1	3	2	1	-3	4	1	3	4	1	3	4	1	3	4
2	1	3	3	-1	3	4	-1	3	4	1	-3	4	-1	-3	4
4	0	3	5	-2	3	4	1	4	4	0	-4	5	-2	-3	5
3	1	3	3	0	0	3	0	0	3	0	3	4	1	3	3
2	-1	3	4	-1	3	4	-1	-3	3	-1	-3	3	-1	-3	3
4	-1	3	2	-1	-3	4	1	3	4	1	0	4	2	4	4
4	-1	3	2	0	3	3	1	3	4	-1	0	4	0	0	4
5	-2	4	1	-2	-4	1	2	4	5	2	4	5	2	4	5

3	1	3	2	-1	0	4	1	3	4	1	0	4	-1	-3	4
5	-2	4	1	-2	-4	5	2	4	5	-2	4	5	2	-4	5
4	0	3	4	1	3	3	1	0	3	0	-3	4	-1	3	4
4	1	3	2	-1	-3	4	1	3	4	-1	3	4	0	3	4
1	-1	0	4	1	0	4	1	3	4	1	-3	4	-1	3	3
4	-1	3	2	-1	-3	3	1	-3	3	0	-3	4	-1	0	4
3	-1	3	4	-1	3	4	1	-3	3	0	0	4	1	0	4
3	0	3	4	1	3	4	-1	-3	2	0	-3	3	-1	0	4
4	0	0	4	0	0	3	1	0	4	-1	-3	3	1	-3	3
3	0	0	3	0	3	5	1	-3	4	-1	-3	4	0	0	5
4	1	4	4	-1	3	5	1	3	3	1	0	4	2	0	4
3	-1	3	5	-1	3	5	-1	-3	5	-2	-4	5	-2	-3	5
3	0	4	5	-2	4	5	1	4	4	-2	-4	5	0	3	4
4	1	3	2	-1	3	4	-1	-3	3	-1	3	4	1	3	3
3	0	3	4	-2	4	3	1	0	4	-1	-3	4	-2	3	4
3	0	4	5	-2	4	5	2	4	4	-2	-3	4	-1	-3	4
4	1	3	2	1	0	4	1	3	3	0	0	5	1	4	5
5	2	4	1	2	-4	5	2	4	5	2	4	5	2	4	4
5	2	0	1	2	-4	4	0	4	3	2	0	4	-2	4	5
4	1	3	2	1	-3	4	1	3	4	1	0	4	0	3	4
2	-1	0	4	-1	3	3	-1	-3	3	0					
2	1	-3	5	-2	3	3	0	0	3	1	-3	3	-2	-4	3
3	-1	0	4	-1	3	3	0	-3	3	1	-3	4	-1	-3	3
3	-1	3	4	-1	3	3	1	0	3	1	-3	4	-1	0	3

2	-1	0	4	-1	3	4	0	-3	4	0	-3	4	-1	-3	4
3	1	3	2	1	-3	4	1	3	4	0	-3	4	0	0	4
3	1	4	1	0	3	4	2	4	3	1	3	5	1	4	5
1	-2	3	1	-1	3	4	1	3	4	-1	0	4	1	0	4
4	2	4	2	1	3	4	1	3	4	1	3	4	0	4	4
	0	0	4	0	3	4	-1	0	4	-2	-4	4	2	3	4
4	1	3	1	2	0	4	1	3	4	-1	3	4	1	4	4
4	0	4	4	1	4	4	2	3	4	-1	3	5	2	4	5
4	1	0	3	1	3	4	1	0	3	0	0	4	1	3	4
3	0	3	2	1	0	4	1	3	3	1	-3	4	-1	4	4
4	2	4	2	0	0	4	1	4	4	1	0	5	1	4	5
3	1	3	4	-1	3	3	1	3	4	0	-3	4	-1	0	4
4	1	3	2	1	0	4	1	3	4	0	-3	4	0	3	4
3	-1	3	3	1	-3	5	1	0	4	-1	-3	4	-1	-3	3
3	0	3	3	-1	3	4	1	0	4	-1	-3	4	0	0	4
3	-1	0	4	-1	3	4	0	0	4	-1	0	4	-1	3	4
3	1	0	4	-1	0	4	0	0	4	-1	-3	4	1	3	4
4	2	3	4	1	-3	4	1	0	4	-1	-3	4	0	3	4
3	0	3	4	-1	3	4	1	0	4	-1	-3	4	-1	3	4
3	-1	3	4	-1	3	4	1	0	4	-1	-3	4	-1	3	4
3	-1	3	4	-2	3	4	1	0	4	-1	-4	4	1	3	4
3	1	3	4	-1	3	4	1	0	4	-1	-3	4	-1	3	4

4		3	3	-1	3	4	1	4	5	-1	0	5	0	0	4

Table S2: FSS raw scores.

FSS_1	FSS_2	FSS_3	FSS_4	FSS_5	FSS_6	FSS_7	FSS_8	FSS_9	Mean score
6	6	6	6	4	4	6	3	6	5.22
7	7	4	4						5.50
7	7	7	7	7	7	7	7	7	7.00
5	6	6	6	6	6	6	6	6	5.89
7	5	5	6	5	5	5	7	5	5.56
7	7	7	7	5	7	6	5	5	6.22
7	7	7	7	7	7	7	7	7	7.00
7		7			7	7	7	7	7.00
7	7	7	5	7	4	7	4	4	5.78
7	7	7	7	7	7	7	7	7	7.00
4	7	4	1	4	4	4	4	4	4.00
7	7	6	6	6	5	6	5	5	5.89
5	6	4	5	5	5	6	5	4	5.00
5	6	4	5						5.00
5	6	4	5	5	5	4	6	5	5.00
6	5	5	5	4	5	5	4	3	4.67
6	6	6	6	6	6	6	6	7	6.11

5	6	6	6	6	6	6	6	5	5.78
4	3	2	2	2	6	5	5	4	3.67
4	1	1	1	1	1	1	1	1	1.33
2	4	5	6	4	7	5	7	7	5.22
5	5	4	3	4	5	3	3	2	3.78
5	6	5	6	5	6	5	6	4	5.33
5	5	5	5	5	6	5	5	5	5.11
5	6	5	6	5	6	6	6	6	5.67
5	6	5	3	3	5	5	2	3	4.11
6	6	6	6	6	7	6	6	6	6.11
6	7	6	6	6	6	6	6	6	6.11
6	6	6	6	6	6	6	6	6	6.00
6	7	6	6	6	6	6	6	5	6.00
6	6	7	6	7	6	6	6	5	6.11
6	6	7	6	6	6	5	6	6	6.00
6	7	7	7	6	7	6	6	6	6.44
5	6	4	5	5	5	4	6	5	5.00
5	5	7	7	4	6	7	4	4	5.44
7	55	7	7	55	7	25	25	1	5.00
5	4	4	2	3	3	6	3	3	3.67
65	65	65	15	15	65	65	65	65	5.39
65	65	65	65	15	35	15	15	15	3.94
5	4	4	4	2	6	3	7	2	4.11
25	1	1	1	1	1	1	1	1	1.17
6	4	4	2	4	4	3	4	4	3.89

65	65	55	65	55	55	65	55	55	5.94
7	4	7	7	7	4	7	7	7	6.33
1	1	1	1	1	1	1	1	1	1.00
5	6	6	5	4	5	5	6	6	5.33
1	5	5	6	4	7	4	1	4	4.11
5	4	4	5	4	3	2	1	2	3.33
6	4	4	4	2	3	2	2	1	3.11
65	65	65	65	65	65	65	65	65	6.50
3	3	5	4	5	5	4	1	1	3.44
6	4	4	6	5	5	3	1	2	4.00
6	4	4	7	5	6	7	6	7	5.78
65	65	65	65	45	65	45	65	35	5.72
6	7	5	7	6	7	6	7	7	6.44
1	1	1	1	1	1	1	1	1	1.00
65	65	65	65	45	65	65	45	65	6.06
15	35	15	15	15	15	15	15	15	1.72
7	4	4	7	7	4	4	4	4	5.00
5	5	5	2	1	2	3	4	4	3.44
7	6	5	5	5	7	6	5	5	5.67
7	7	7	7	7	7	7	7	7	7.00
7	7	4	4	7	7	7	7	7	6.33
5	5	5	6	4	6	4	3	2	4.44
5	5	6	6	5	4	3	2	4	4.44
5	5	1	1	1	4	1	1	1	2.22
7	7	7	7	4	4	7	7	4	6.00

1	1	1	1	1	1	1	1	1	1.00
5	4	1	1	1	2	1	1	1	1.89
65	45	45	45	65	5	45	45	45	5.00
65	65	65	45	45	65	45	65	45	5.61
65	65	65	65	65	65	65	65	65	6.50
65	65	65	65	65	65	65	65	65	6.50
5	5	5	5	5	5	5	5	5	5.00
5	5	5	6	5	5	5	5	5	5.11
2	2	2	2	2	2	2	2	2	2.00
2	3	3	3	2	2	2	2	2	2.33
3	3	3	3	3	3	3	3	3	3.00
5	4	5	5	5	5	5	5	5	4.89
3	3	3	3	3	3	3	3	3	3.00
5	4	5	4	5	5	5	5	5	4.78
6	6	5	5	5	5	6	6	6	5.56
5	5	5	5	5	6	5	6	5	5.22
2	2	2	2	2	2	2	2		2.00
3	3	3	3	3	3	3	3	3	3.00
5	5	5	6	5	5	6	6	5	5.33
5	5	5	5	5	5	5	5	5	5.00
5	5	5	5	5	5	5	5	5	5.00
3	2	2	2	2	2	2	2	2	2.11
6	6	6	6	6	6	6	6	6	6.00
1	1	1	1	1	1	1	1	1	1.00
5	5	5	5	6	5	6	5	6	5.33

2	2	2	1	1	2	2	2	2	1.78
5	5	5	5	5	5	5	6	6	5.22
3	3	3	4	5	5	5	4	5	4.11
2	2	1	2	2	2	2	2	2	1.89
2	2	2	2	2	2	2	2	2	2.00
2	2	2	2	2	2	2	3	3	2.22
3	2	3	2	2	3	3	2	2	2.44
5	5	5	5	5	5	6	5	5	5.11
55	4	45	55	35	45	45	55	4	4.61
6	4	7	5	6	3	5	6	4	5.11
6	6	7	6	6	6	6	6	6	6.11
6	3	3	2	2	3	2	2	2	2.78
7	4	4	7	4	7	6	1	4	4.89
6	6	6	6	4	6	4	5	6	5.44
5	6	3	4	3	3	3	3	3	3.67
6	6	6	6	4	6	2	6	2	4.89
2	2	2	2	3	3	3	3	3	2.56
5	5	5	5	5	5	5	5	5	5.00
5	5	5	5	4	4	5	4	5	4.67
5	5	5	5	5	6	5	5	5	5.11
5	5	5	5	4	5	5	5	5	4.89
5	5	5	5	5	5	5	5	5	5.00
3	3	3	3	3	3	3	3	3	3.00
3	3	3	3	3	3	3	3	3	3.00
1	1	1	1	1	1	1	1	1	1.00

6	6	6	6	6	6	6	6	6	6.00
2	2	3	3	2	2	2	3	2	2.33
5	5	5	5	5	5	6	5	6	5.22
3	3	3	3	3	3	3	3	3	3.00
6	6	6	6	6	6	6	6	6	6.00
3	3	3	3	3	3	3	3	3	3.00
3	3	3	3	3	3	3	3	3	3.00
6	6	6	6	6	6	6	6	6	6.00
3	2	3	3	3	3	3	3	3	2.89
6	6	5	5	5	6	5	6	5	5.44
4	5	5	5	4	5	5	5	5	4.78
5	5	5	5	5	5	5	5	5	5.00
3	3	3	3	3	3	3	3	3	3.00
5	5	5	5	5	5	5	5	5	5.00
5	5	5	5	5	5	5	5	5	5.00
5	5	5	5	5	5	5	5	5	5.00
3	3	3	3	3	3	3	3	3	3.00
5	5	5	5	5	5	5	5	5	5.00
5	5	5	5	5	5	5	5	5	5.00
3	3	3	3	3	3	3	3	3	3.00
5	5	5	5	5	5	5	5	5	5.00
1	1	1	1	1	1	1	1	1	1.00
2	2	2	2	2	2	2	2	2	2.00
5	5	5	5	5	5	5	5	5	5.00
5	5	5	5	5	5	5	5	5	5.00

2	2	2	2	2	2	2	2	2	2.00
3	3	3	3	3	3	3	3	3	3.00
5	5	5	5	5	5	5	5	5	5.00
3	3	3	3	3	2	3	2	3	2.78
5	5	5	5	6	5	5	5	5	5.11
3	3	3	4		3	3	2		3.00
4	4	4	4	4	4	4	4	4	4.00
5	5	4	5	5	5	5	5	5	4.89
3	4	3	4	3	3	3	3	3	3.22
3	3	3	3	3	3	3	2	3	2.89
2	2	2	2	2	2	2	2	2	2.00
3	4	3	3	3	5	5	5	3	3.78
2	2	2	2	2	2	2	2	2	2.00
5	5	5	5	5	5	5			
